# Supplementary material for: High Glucose Promotes Inflammation and Weakens Placental Defenses against E. coli and S. agalactiae Infection: Protective Role of Insulin and Metformin
Source: Int J Mol Sci. 2023 Mar 9;24(6):5243. doi: 10.3390/ijms24065243 (PMC10048930; doi:10.3390/ijms24065243)
Supplement: Supplementary file 1 [file ijms-24-05243-s001.zip › ijms-2195527-si.pdf]

Supplementary Table S1. Osmolarity in supplemented media culture adjusted with different glucose and mannitol concentrations

Treatment	mOsm	Osmolarity should be equivalent to
**5 mM glucose**	316	
**10 mM glucose**	320	
**50 mM glucose**	353	
**5 mM glucose + mannitol 5 mM**	320	10 mM glucose
**10 mM glucose + mannitol 40 mM**	346	50 mM glucose

Media base was DMEM low-glucose + 10% FBS + 1% sodium pyruvate + 1% penicillin/streptomycin

Supplementary Table S2. Placental adipokine secretion under normoglycemic and hyperglycemic conditions.

	Visfatin	Chemerin	Adiponectin	Leptin
**Glucose 10 mM**	10,248<br>(8,648 – 13,460)	9,297<br>(5,689 – 17,767)	28,007<br>(20,867 – 47,727)	425.1<br>(297.9 – 652.1)
**Glucose 50 mM**	7,749<br>(5,459 – 9,972) #	10,578<br>(7,857 - 18,209)	28,781<br>(19,823 - 51,499)	435<br>(270.5 - 799.3)
**Glucose 10 mM<br>+ Insulin 500 nM**	13,710<br>(9,978 - 19,128)	10,140<br>(7,604 - 16,828)	23,888<br>(16,372 - 39,535)	379<br>(271.7 - 652.1)
**Glucose 50 mM<br>+ Insulin 50 nM**	3,159<br>(1,972 - 5,317) *	12,278<br>(8,234 - 19,831)	21,039<br>(15,055 - 38,694)	492.9<br>(329.4 - 715)
**Glucose 50 mM<br>+ Insulin 100 nM**	4,327<br>(2,999 - 8,064) *	11,881<br>(8,804 - 16,323)	26,684<br>(20,236 - 50,233)	405.7<br>(312.1 - 608)
**Glucose 50 mM<br>+ Insulin 500 nM**	4,058<br>(2,191 - 6,727) *	11,980<br>(8,086 - 18,622)	33,429<br>(22,327 - 53,150)	343.2<br>(229 - 511.7)
**Glucose 10 mM<br>+ Metformin 500**	11,777<br>(8,575 - 14,692)	6,187<br>(3,381 - 12,603)	27,641<br>(18,569 - 48,020)	410.6<br>(329 - 756.7)
**Glucose 50 mM +<br>Metformin 125**	8,893<br>(5,200 - 12,345)	9,240<br>(4,345 - 15,842)	34,539<br>(19,751 - 55,366)	334.9<br>(240 - 495.4)
**Glucose 50 mM +<br>Metformin 250**	6,348<br>(3,605 - 9,766)	7,714<br>(5,211 - 14,340)	27,952<br>(20,368 - 63,250)	618.9<br>(265.8 - 838.6)
**Glucose 50 mM<br>+ Metformin 500**	6,171<br>(3,084 - 9,905)	8,871<br>(4,611 - 15,371)	35,553<br>(20,667 - 60,592)	334.9<br>(211.6 - 465.5)

Visfatin, chemerin, leptin, and adiponectin levels are shown in pg/mL/ g of tissue. #, $p < 0.05$ vs Glucose 10 mM. *, $p < 0.05$ vs Glucose 50 mM. n = 6 – 7 independent experiments in triplicate. One-way ANOVA (Kruskal-Wallis test) followed by Dunn´s multiple comparisons post-hoc. Data is presented as median (interquartile range).

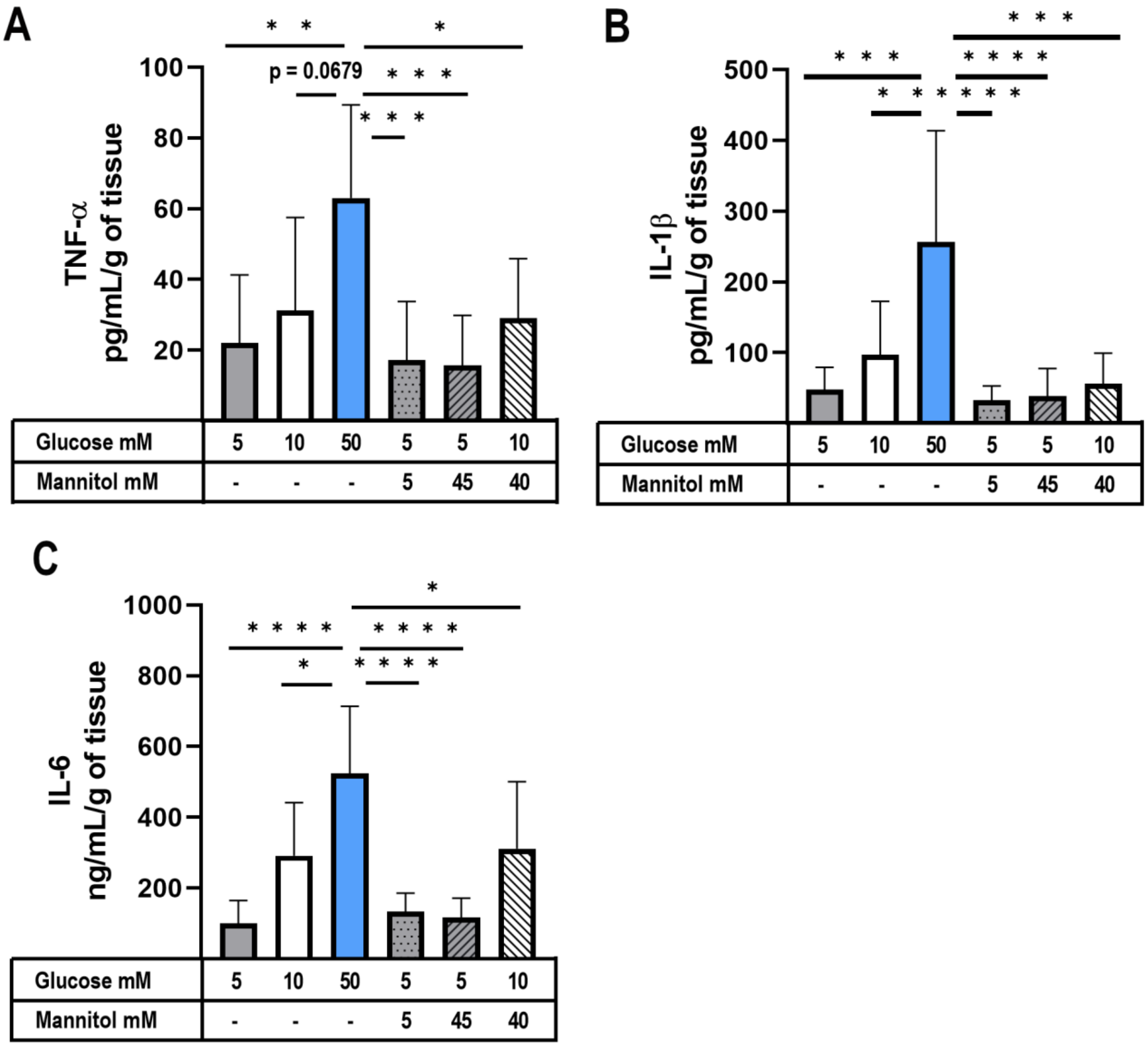

Supplementary Figure S1. High osmolar pressure does not increase secretion of pro-inflammatory cytokines in cultured placental explants. Placental explants secretion of A) TNF-α, B) IL-1β, and C) IL-6. Explants were exposed to glucose and co-incubated with mannitol for 48 hours. Treatments and culture media were refreshed at 24 hours. n = 3 independent experiments in triplicate. Data are presented as mean and standard deviation. One-way ANOVA followed by Tukey's multiple comparisons post-hoc due to normal distribution *, $p < 0.05$; **, $p < 0.01$; ***, $p < 0.001$; ****, $p < 0.0001$

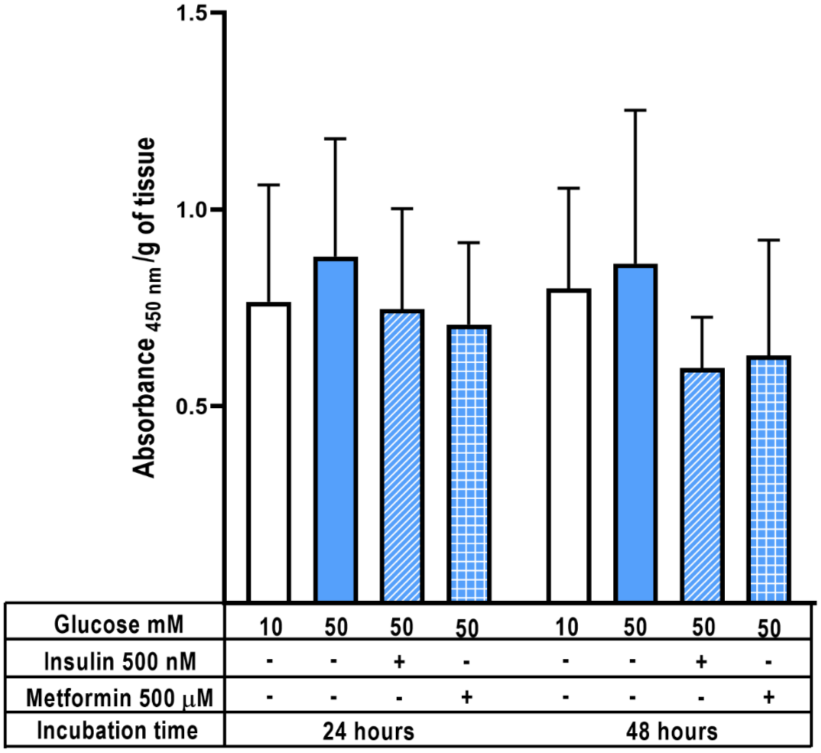

Supplementary Figure S2. Insulin and metformin do not modify viability of placental explant along 48 hours of culture. XTT cell viability assay of placental explants incubated along 48 hours with culture media adjusted with glucose 10 mM (white bar) or 50 mM (blue bar). n = 3 independent experiments in triplicate. Two-way ANOVA (mixed effects analysis) followed by Tukey´s multiple comparisons test. No significant differences were detected. Data is presented as mean and standard deviation.
